# NIR‐II Fluorescent Thermophoretic Nanomotors for Superficial Tumor Photothermal Therapy

**DOI:** 10.1002/adma.202417440

**Published:** 2025-02-02

**Authors:** Jiwei Jiang, Jing Hu, Mingtong Li, Mingzhi Luo, Bin Dong, Metin Sitti, Xiaohui Yan

**Affiliations:** ^1^ Institute of Functional Nano & Soft Materials (FUNSOM) Jiangsu Key Laboratory for Carbon‐Based Functional Materials & Devices Soochow University Suzhou 215123 China; ^2^ State Key Laboratory of Vaccines for Infectious Diseases Center for Molecular Imaging and Translational Medicine Xiang An Biomedicine Laboratory School of Public Health Xiamen University Xiamen 361005 China; ^3^ Physical Intelligence Department Max Planck Institute for Intelligent Systems 70569 Stuttgart Germany; ^4^ Changzhou Key Laboratory of Respiratory Medical Engineering Institute of Biomedical Engineering and Health Sciences School of Medical and Health Engineering Changzhou University Changzhou Jiangsu 213164 China; ^5^ School of Medicine and College of Engineering Koç University Istanbul 34450 Turkey

**Keywords:** micro‐/nanomotors, NIR‐II imaging, peritumoral administration, photothermal therapy, subcutaneous delivery

## Abstract

Peritumoral subcutaneous injection has been highly envisioned as an efficient yet low‐risk administration of photothermal agents for superficial tumor photothermal therapy. However, obstructed by complex subcutaneous tissue, the delivery of injected photothermal agents to the specific tumor remains a critical issue. Herein, the study reports a polydopamine (PDA)‐encapsulated spherical core/shell nanomotor with fluorescent indocyanine green (ICG) immobilized on its PDA shell. Upon the first near‐infrared (NIR‐I) irradiation, this motor can generate favorable photothermal heat, and meantime, emit a robust ICG fluorescence in the second near‐infrared window (NIR‐II). The heat turns the motor into an active photothermal agent able to perform thermophoretic propulsion along the irradiation direction in subcutaneous tissue, while the ICG fluorescence can direct the subcutaneous propulsion of motors toward specific tumor through real‐time NIR‐II imaging. These functions endow the motor with the ability of moving to tumor after being injected at peritumoral site, enabling an enhanced photothermal therapy (PTT). The results demonstrated herein suggest an integrated nanorobotic tool for the superficial PTT using peritumoral administration, highlighting an NIR‐II imaging‐directed subcutaneous propulsion.

## Graphical abstract

A spherical core‐shell nanomotor consisting of a polydopamine‐indocyanine green composite shell and a rigid functional core is proposed in this work. This motor can be activated by the NIR‐I irradiation to perform NIR‐II imaging‐directed thermoplastic propulsion in subcutaneous tissue, enabling an efficient peritumoral administration for low‐risk superficial tumor photothermal therapy.

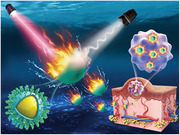

## 1 Introduction

Superficial malignant tumors, such as melanoma, basal cell carcinoma, and squamous cell carcinoma, have been at high incidence rate in the past decade.^[^ ^1^, ^2^, ^3^, ^4^ ^]^ These tumors are susceptible to metastasis and feature poor prognosis, which seriously compromises the health and life quality of patients.^[^ ^5^, ^6^, ^7^ ^]^ In clinical practices, surgical resection has been used as the most common therapeutic modality for them. It is a traumatic method featured with long recovery time, and also, may affect the patient's appearance after recovery from surgical trauma.^[^ ^8^ ^]^ Furthermore, the patients being in severe diabetes, immunodeficiency, and other conditions unsuitable for surgery are unable to use such treatment.^[^ ^1^, ^9^ ^]^ Therefore, the development of noninvasive therapeutic modalities, such as photothermal therapy (PTT), topical radiotherapy, chemotherapy, and immunotherapy, remains highly desired for superficial tumor treatment.^[^ ^10^, ^11^ ^]^ The PTT, one topical treatment relying on near‐infrared (NIR) light, could be favorable since it avoids the adverse reactions probably arisen from chemotherapy and immunotherapy, and at the same time, eliminates the radiation hazard of radiotherapy.^[^ ^12^, ^13^, ^14^ ^]^ To date, this advantage of PTT has been clarified in the treatment of basal cell carcinoma and melanoma.^[^ ^12^, ^15^ ^]^

To enhance the anti‐tumor efficacy of PTT, photothermal agents that can convert near‐infrared (NIR) light energy into physical heat are needed.^[^ ^12^, ^13^, ^14^, ^15^, ^16^ ^]^ Systemic, intertumoral, and peritumoral injection are three major approaches used for the administration of photothermal agents.^[^ ^17^ ^]^ The administration using systemic injection relies on blood circulation, primarily achieved through intravenous injection and intraperitoneal injection.^[^ ^18^, ^19^ ^]^ In this strategy, only a small proportion of photothermal agents can be delivered to those designated tumors as blood flow. The remaining majority are enriched in health tissues and organs, which probably bring serious side effects. By contrast, intertumoral injection as a localized administration method can directly inject the chosen photothermal agents to specific tumor with the aid of mechanical extrusion, and therefore, improve the issues of biosafety and inefficiency existing in systemic administration.^[^ ^20^, ^21^ ^]^ However, due to the usage of mechanical extrusion, intertumoral injection exhibits a high risk of disrupting the tumor vasculature and causing tumor cell metastasis.^[^ ^22^ ^]^ Peritumoral injection also is a topical administration. Unlike the intertumoral injection, this administration method only injects photothermal agents into subcutaneous tissue at suitable sites near tumor.^[^ ^23^ ^]^ Such operation can eliminate the risk of tumor cell metastasis and shorten the delivery distance of photothermal agents when compared to the systemic administration.^[^ ^24^, ^25^, ^26^ ^]^ Attributed to these advantageous attributes, peritumoral injection has been explored as an efficient yet low‐risk administration for superficial tumor PTT.

Notwithstanding considerable advances achieved by the peritumoral administration for superficial tumor PTT, the subcutaneous delivery of injected photothermal agents to designated tumor remains a challenge. Current strategies accessible for the subcutaneous delivery largely rely on the concentration gradient‐driven passive diffusion,^[^ ^27^, ^28^, ^29^ ^]^ in which all injected photothermal agents spread around the injection sites with low diffusion rates. Without the active motion and directed ability, these strategies are difficult to gain a high efficiency. Micro/nanomotors, extensively explored for efficient cargo delivery in the past two decades, may offer a promising strategy for delivery after peritumoral injection.^[^ ^30^, ^31^ ^]^ Powered by external physical fields (e.g., light, ultrasound, magnetism, and electricity), chemical/enzymatic reaction, and biological energy, they can perform different modes of motion in diverse biological environments.^[^ ^26^, ^32^, ^33^, ^34^, ^35^, ^36^, ^37^, ^38^, ^39^, ^40^, ^41^, ^42^, ^43^, ^44^, ^45^, ^46^, ^47^, ^48^ ^]^ The motion, also combining enzymatic effect in some cases, has endowed micro/nanomotors with a robust ability to overcome multiple biological barriers including complex subcutaneous tissue (major obstruction for the subcutaneous delivery, mainly composed of adipose cells and connective protein networks).^[^ ^26^, ^39^, ^40^, ^41^, ^42^, ^43^, ^44^, ^45^, ^46^, ^47^, ^48^, ^49^ ^]^ This ability makes the motors feasible for active delivery in subcutaneous tissue. Furthermore, by integrating suitable control methods, the motion of micro‐/nanomotors can be easily guided,^[^ ^26^, ^40^, ^43^, ^44^ ^]^ which guarantees the motor‐enabled active delivery mostly toward the designated tumor. The functions demonstrated above suggest that development of micro/nanomotors that are able to penetrate complex subcutaneous tissues and perform controlled motion is highly desired for efficient delivery after peritumoral injection.

**Figure 1:**
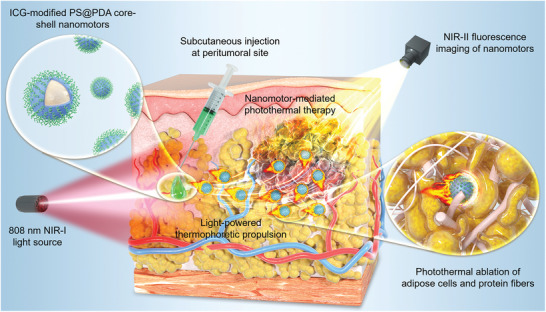
Schematic illustration of the nanomotor‐dominant tumor photothermal therapy. The motor consists of an ICG‐modified PDA shell and a spherical PS core, in which the core could be replaced with other materials as needed. In this therapeutic modality, a swarm of the motors is injected to the subcutaneous tissue near a specific tumor (i.e., at peritumoral regions) and irradiated by an 808 nm NIR‐I light along the designated direction. Upon the irradiation, those injected motors generate photothermal heat and a NIR‐II fluorescence at the same time. The heat liquefies adipose cells (one major component of the subcutaneous tissue) surrounding the motor and damages connective protein network (the other major component of subcutaneous tissue), making a thermophoretic propulsion toward the irradiation direction. The whole propulsion can be monitored and directed using NIR‐II fluorescence through real‐time NIR‐II imaging. This performance enables a precise and efficient delivery of the motor to the tumor. At the tumor site, the motor can use the heat to achieve desired photothermal therapy.

In this work, we propose a polydopamine (PDA)‐encapsulated spherical nanomotor with indocyanine green (ICG, a fluorescent molecule approved by FDA for clinical uses) anchored on the PDA shell (**Figure** **1**). The PDA and ICG are active components, while the core mainly acts as a carrier of PDA and ICG and serves for propulsion. From a propulsion point of view, the core could be any materials with spherical shape and certain rigidity.^[^ ^41^, ^42^ ^]^ Herein, a polystyrene (PS) nanosphere featuring low cost, good dispersibility, easy purchase/fabrication, and uniform particle size, is chosen as the core of our proposed motor (a PDA‐encapsulated PS nanosphere nanomotor immobilized with ICG, termed as PS@PDA‐ICG hereafter) for conceptual demonstration. Benefited from the co‐existence of PDA and ICG, the PS@PDA‐ICG naturally is a fluorescent photothermal agent. Upon an irradiation from the first NIR window (NIR‐I), it can generate heat and meantime emit a strong fluorescence in the second NIR window (NIR‐II). The heat amount attenuates along the irradiation direction, creating a directional thermophoretic force for propulsion. Furthermore, the heat can ablate adipose cells and connective protein networks, both of which are the major component of subcutaneous tissue. This effect decreases the resistance of complex subcutaneous tissue and enables a directed propulsion in model mice. On the other hand, the NIR‐II fluorescence allows real‐time NIR‐II imaging, which can be used to monitor and guide the propulsion toward specific tumor. Equipped with these functions, the motor accomplishes an animal superficial PTT trial via peritumoral administration, demonstrating an integrated active nanoplatform for efficient yet low‐risk PTT.

## 2 Results

### 2.1 Fabrication and Performance Characterization

**Figure 2:**
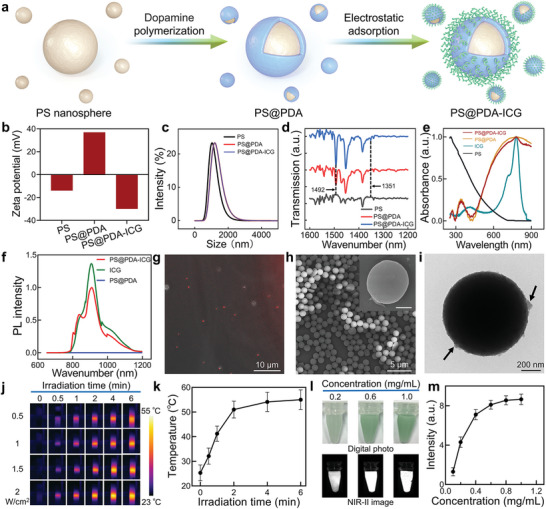
Fabrication and characterization of the proposed PS@PDA‐ICG nanomotor. a) Schematic of the fabrication process. b) Zeta potentials of the core material (PS nanosphere), intermediate product (PDA‐encapsulated PS nanosphere, PS@PDA), and final product (the nanomotor, PS@PDA‐ICG). c,d) Hydrated particle size distribution and Fourier transform infrared (FTIR) spectra of PS, PS@PDA and PS@PDA‐ICG. e) UV–vis–NIR spectra of ICG, PS, PS@PDA and PS@PDA‐ICG. f) Fluorescence spectra of PS@PDA, ICG and PS@PDA‐ICG. The excitation wavelength is 808 nm. g) Confocal laser scanning microscopy image of PS@PDA‐ICG in the NIR‐II window. h) SEM images of PS@PDA‐ICG. The inset (scale bar: 500 nm) is an enlarged SEM image for a single PS@PDA‐ICG. i) TEM image of PS@PDA‐ICG. The arrows indicate the PDA and ICG on the surface. j) Time‐lapse thermal images of 1 mg/mL PS@PDA‐ICG subject to 808 nm NIR‐I irradiation at varying power. k) Photothermal heating curve extracted from (j). l) Digital photos and NIR‐II images of PS@PDA‐ICG at varying concentration. m) NIR‐II fluorescence intensity as a function of concentration. The intensity is read from (l) and Figure S4 with the ImageJ software. The error bars in (k) and (m) are the standard deviations calculated from three experiments.

The proposed PS@PDA‐ICG is fabricated via a two‐step process consisting of dopamine self‐polymerization or PDA deposition on PS nanospheres and ICG immobilization to the deposited PDA through an electrostatic adsorption (**Figure** **2**a). To confirm the successful fabrication of PS@PDA‐ICG, multiple characterizations have been carried out. The result of Zeta potential testing is shown in Figure 2b, wherein the potential changes from the ‐14 mV of PS to the 37 mV and ‐30 mV of the intermediate product and final product. Along with the change of Zeta potential, a larger hydrated size is also measured in intermediate and final products (Figure 2c). Furthermore, in the Fourier transform infrared (FTIR) spectra (Figure 2d), the final product shows the characteristic peak of PDA (1492 cm^−1^, corresponding to the N‐H shearing vibration of amide group) and ICG (1351 cm^−1^, corresponding to the S = O stretching vibration of sulfonyl group). Additionally, we find two peaks at 345 and 785 nm in the UV–vis–NIR adsorption spectrum of final product (Figure 2e, red curve). These two peaks can be corresponded to the characteristic absorption of PDA (Figure 2e, yellow curve) and ICG (Figure 2e, green curve). All these results, brought together, suggest that the final product is PS@PDA‐ICG and thus a successful fabrication.

In the fabricated PS@PDA‐ICG, the weight percentage of PDA and ICG is estimated to be 37.36% and 0.88% (see the Experimental Section for detailed measurement and calculation). The immobilized ICG exhibits a fluorescence emission spectrum akin to the freely suspended ICG (Figure 2f), suggesting that the PS@PDA‐ICG inherits the NIR‐II fluorescence of ICG. And the ICG immobilization should be homogenous on the PS@PDA‐ICG, as indicated by the fluorescence distribution in a confocal laser scanning microscopy (CLSM) image (Figure 2g). The fluorescence will be highlighted for a real‐time NIR‐II imaging and such imaging‐directed propulsion in subcutaneous tissue. As depicted by scanning electron microscopy (SEM) image, the PS@PDA‐ICG features the uniform size, good dispersibility, and spherical shape of the PS matrix (Figures 2h and S1, Supporting Information). These advantageous attributes are crucial for the following propulsion and biomedical applications. Further characterization using transmission electron microscopy (TEM) imaging finds that the PS@PDA‐ICG possesses a rough surface (Figure 2i). This morphology should be caused by the PDA‐ICG composite since it is not observed on the PS surface (Figure S2, Supporting Information). Besides, with the help of SEM imaging, we have verified that the PS core can be replaced with other functional materials, such as biodegradable silica, magnetic nanoparticle and polycaprolactone (Figure S3, Supporting Information).

The emergence of NIR region absorption in Figure 2e indicates that the PS@PDA‐ICG can absorb the NIR light and convert it into physical heat.^[^ ^50^, ^51^, ^52^ ^]^ Therefore, we measured the photothermal performance of PS@PDA‐ICG. Under 1.5 W cm^−2^ 808 nm NIR‐I irradiation, the temperature of 1 mg mL^−1^ PS@PDA‐ICG suspension can increase to 55 °C in 2 min (Figure 2j,k), which could be notable for tumor PTT. Next, we tested the performance of PS@PDA‐ICG's fluorescence for imaging using a commercial in vivo NIR‐II imaging system. Imaging signals can be detected in the PS@PDA‐ICG samples of different concentration (Figures 2l and S4, Supporting Information). By comparing the NIR‐II images of PS, PS@PDA and ICG, the above signals can be easily verified to be from the NIR‐II fluorescence of ICG (Figure S5, Supporting Information). Quantitative analysis finds that the signal intensity can gradually rise to reach a leveling off stage as the concentration increased (Figure 2m). This result shows an existence of fluorescence quenching phenomena in the PS@PDA‐ICG suspension.

### 2.2 Photo‐Thermophoretic Motion and NIR‐II Imaging‐Based Tracking

**Figure 3:**
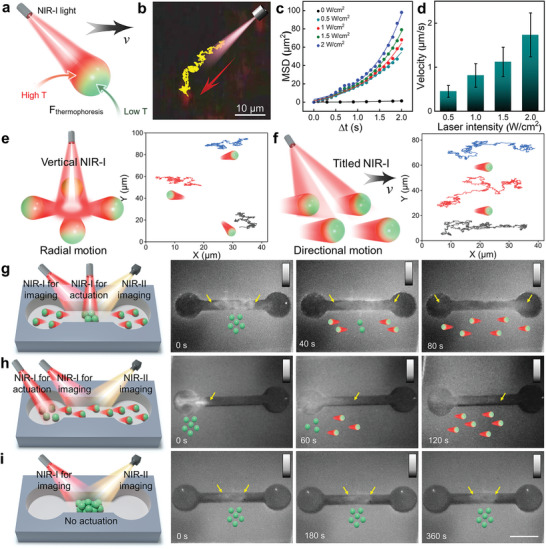
Photo‐thermophoretic motion of the PS@PDA‐ICG nanomotor and the motion‐related NIR‐II imaging. a) Schematic demonstration of the thermophoretic mechanism. b) Motion trajectory of a PS@PDA‐ICG in PBS upon the irradiation with 1.5 W cm^−2^ 808 nm light for 30 s. c) velocity of the PS@PDA‐ICG at different laser intensity (or irradiation power). Error bars shown herein are the standard deviation of three independent experiments. d) Mean square displacement (MSD) versus time interval at different irradiation power. e,f) Schematic and measured motion trajectory for the thermophoretic propulsion of multi‐body nanomotors upon the vertical and tilted NIR‐I irradiation (1.5 W cm^−2^). g–i) Schematic and NIR‐II image sequence for in vitro swarm propulsion in a microchannel under a vertical irradiation and tilted irradiation and no extra irradiation. The yellow arrows indicate the boundary of signal shifting. All NIR‐II images of (g–i) share the same scale bar, 5 mm. In the schematic, these green balls represent the PS@PDA‐ICG.

The photothermal effect of PDA and ICG leads to the heat generation of the PS@PDA‐ICG irradiated by an 808 nm NIR‐I light. As the PS@PDA‐ICG naturally is opaque, the shading effect always exists when a directional NIR‐I light is exerted to the motor. This effect can result in the heat attenuation along the direction of light irradiation (**Figures** **3**a andS6, Supporting Information), which creates a thermophoretic force. Attributed to the force,^[^ ^53^ ^]^ the PS@PDA‐ICG is propelled along the direction of light irradiation in a phosphate buffered saline (PBS) solution (Figure 3b; Movie S1, Supporting Information). In the case without exerting the irradiation, the nanomotor only exhibits a Brownian motion (the 0 W/cm^2^ group of Figures 3c and S7a, Supporting Information), further clarifying that the propulsion is dominated by photothermal effect. High irradiation intensity can bring strong photothermal effect, and therefore, significantly improve the velocity and mean square displacement (MSD) of nanomotor (Figures 3c,d and S7a–d, Supporting Information). Meanwhile, the motor's composition can also control its motion behaviors via affecting the photothermal effect. In this work, the introduction of ICG substantially enhances the photothermal effect of nanomotor (Figure S8, Supporting Information) and brings a favorable motion behavior (Figure S9, Supporting Information). Furthermore, in our tested samples, we find that their motion behaviors are positively correlated with the motor size (Figure S10, Supporting Information). This phenomenon should be attributed to the stronger shadow effect caused by a larger size.

The multi‐body motion of nanomotors has been examined in PBS solution with an optical microscope. As presented in Figure 3e,f, the tested nanomotors can be actuated at the same time and move away from the irradiation. For detailed information about the real‐time motion, please see Movies S2 and S3 (Supporting Information). Next, we demonstrate the collective behaviors of nanomotors with a real‐time NIR‐II imaging. All tested samples were added to the middle of a microchannel placed in a commercial NIR‐II imaging system (Figure 3g–i). In the case of applying tilted and vertical irradiation for propulsion, it can be easily found that the NIR‐II signals of samples, akin to the multibody motion, extend along these directions away from the irradiation (Figure 3g,h). The same or similar signal shifting is not observed in the case without applying any irradiation for propulsion (Figure 3i). These results exclude the influences (e.g., the Brownian motion) from the imaging irradiation, verifying that the signal shifting is merely caused by the external irradiation‐controlled swarm motion. In other words, the signal shifting represents the swarm motion of our motors and meantime the swarm motion of our motors can be monitored through the real‐time NIR‐II imaging. According to the patterns of NIR‐II signal shifting, the swarm motion equivalent to the multi‐body motion is also along the direction away from the irradiation.

As aforementioned, the direction of the motor motion can be controlled by the irradiation. Attributed to the shading effect of motor, the irradiation through photothermal effect creates a thermophoretic force toward the direction of irradiation. Theoretically, the motor should move along the direction of the thermophoretic force (i.e., the irradiation direction). In experiments, our motor propulsion was carried out on a glass and an intact polymer microchannel that the motor cannot penetrate. It hinders the movement along the irradiation and makes the movement toward the directions with less resistance. This effect, together with the thermophoretic force, cause that the motor moves away from the irradiation in Figure 2e–h.

### 2.3 In Vivo Subcutaneous Propulsion

**Figure 4:**
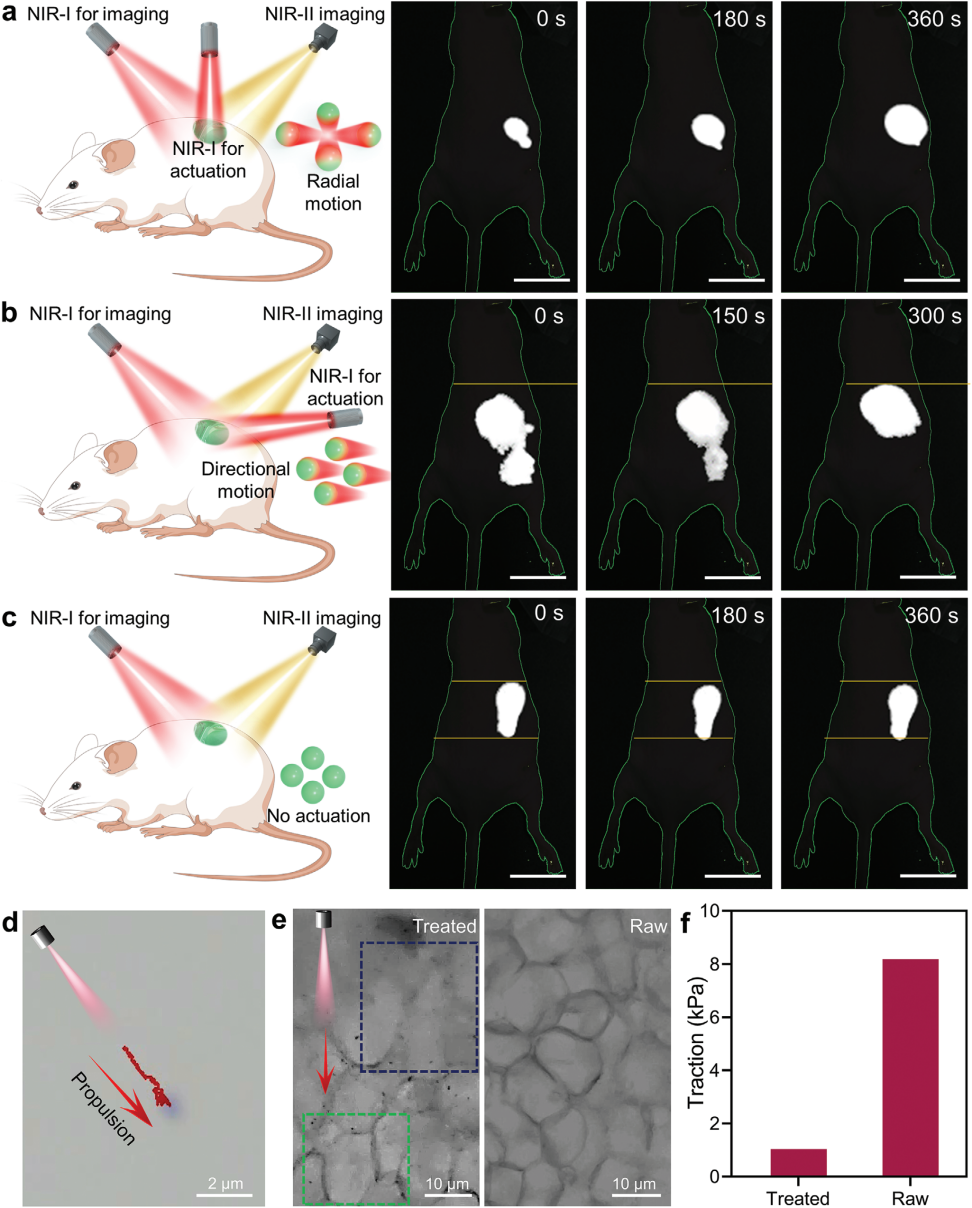
In vivo subcutaneous thermophoretic propulsion and related mechanism. a–c) Schematic and NIR‐II image sequence of the propulsion with a vertical NIR‐I irradiation, a tilted NIR‐I irradiation, and no extra irradiation. d) Motion trajectory of an individual motor propelled in an agarose hydrogel. e) Optical images of a treated and raw subcutaneous tissue collected from a chicken leg. f) Traction of the raw and treated subcutaneous tissues.

On the basis of the results obtained from in vitro swarm propulsion and NIR‐II imaging, we explore the in vivo subcutaneous propulsion of our motor. The related characterization used the in vitro experimental setup except that the microchannel was replaced with model mice (**Figure** **4**a‐c, the schematic). As suggested by the NIR‐II images of each group, the signal movement versus irradiation time is akin to that of in vitro propulsion (Figure 4a–c). These results show that our motors can be propelled in mouse subcutaneous tissue along the direction away from the irradiation, enabling the delivery to tumor after a peritumoral administration. Interestingly, the signal intensity extracted from Figure 4a (i.e., the group with vertical irradiation) increases as the irradiation time prolonged (Figure S11, Supporting Information) and the signal from Figure 4c (i.e., the group without using extra irradiation) is stable (Figure S12, Supporting Information). According to the intensity‐concentration relation presented in Figure 2m, we ascribe this phenomenon to the reduction or elimination of the concentration‐induced fluorescence quenching by swarm propulsion.

To further clarify the subcutaneous propulsion of our motors, we performed experiments in an agarose hydrogel. Here the hydrogel was chosen to mimic the subcutaneous tissue.^[^ ^54^, ^55^, ^56^ ^]^ With an optical microscope, the movement of an individual motor can be clearly observed and such movement is toward the direction away from irradiation (Figure 4d; Movie S4, Supporting Information), verifying the results of the above NIR‐II imaging. We also performed an ex vivo propulsion in a subcutaneous tissue separated from a fresh chicken leg. Note that the chicken leg was purchased from the market. The results are shown in Figure 4e. In comparison with raw tissue, the melting of subcutaneous tissue (adipose cells and protein fibers) and some motors (the high‐contrast black dots) can be determined in the tissue subject to the propulsion. The irradiation for propulsion cannot cause such melting (Figure S13, Supporting Information). With the exclusion of this factor, we believe that the melting should be attributed to the photothermal effect of motor.^[^ ^55^, ^56^ ^]^ The melting creates a liquid environment and reduces the traction of subcutaneous tissue (Figure 4f), enabling the motor movement. The melted tissue, together with intact muscle underneath the subcutaneous tissue, constitutes an environment akin to the aforementioned microchannel and hence the in vivo motors also move as the patterns of in vitro propulsion.

### 2.4 NIR‐II Imaging‐Directed Photothermal Therapy

**Figure 5:**
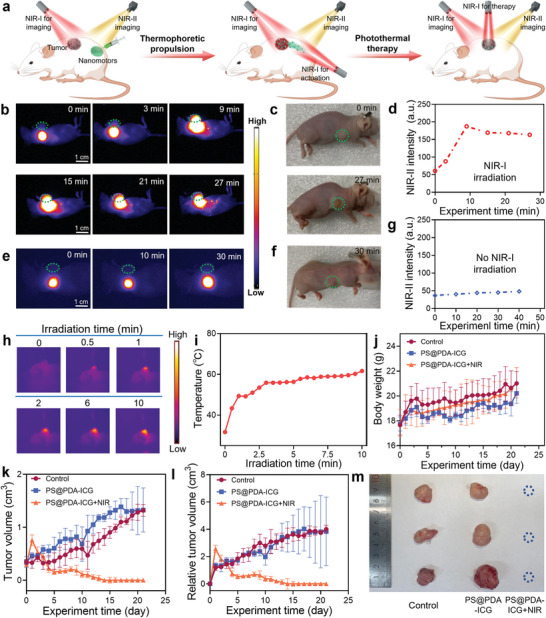
Therapeutic trials of the PS@PDA‐ICG nanomotors in model melanoma tumor mice. a) Schematic of a therapeutic process using the PS@PDA‐ICG. b) Time‐lapse NIR‐II images captured in the subcutaneous delivery of a PS@PDA‐ICG swarm to melanoma tumor. c) Tumor digital photos captured before and after the delivery operation (or light‐powered propulsion). d) Changes of the NIR‐II fluorescence intensity of tumor site in the delivery process. The tumor is highlighted by dotted green circle shown in (b), and the intensity is directly read from (b) with the ImageJ software. e) Time‐lapse NIR‐II images of an injected PS@PDA‐ICG nanomotor swarm in the experiment without using irradiation for actuation. f) Tumor digital photo captured from the experiment without using irradiation for actuation. g) Time‐varying NIR‐II fluorescence intensity of the tumor site selected by dotted green circle in (e). h,i) Thermal imaging and related quantitative analysis for the model tumor subject to 808 nm NIR‐I irradiation after achieving subcutaneous delivery. j) Changes of mice body weight as time in different treatment. k,l) Tumor growth curves after mice subject to different treatment. m) Photographs of the tumors collected from different experimental groups after accomplishing photothermal treatment. Error bars in (j–l) represent the standard deviation of three independent experiments.

After being verified to have the functions of performing in vivo subcutaneous propulsion and NIR‐II imaging, the PS@PDA‐ICG is ready for a tumor PTT trial. The experiments will be carried out in the mice bearing melanoma, including peritumoral injection, subcutaneous delivery to tumor and tumor PTT (**Figure** **5**a). Before conducting the animal experiments, the cytotoxicity of PS@PDA‐ICG is characterized using the 3‐(4,5‐dimethylthiazol‐2‐yl)‐2,5‐diphenyltetrazoliumbromide (MTT) assays. The cell viability remains about 90% even at a high concentration of 600 µg mL^−1^ (Figure S14, Supporting Information), and hence, the PS@PDA‐ICG features low cytotoxicity. The function of PTT is also explored in vitro with a tumor spheroid as the test model. A large number of dead cells are found in the PTT group (Figure S15, Supporting Information), suggesting the feasibility of the PTT using the PS@PDA‐ICG.

Based on the above results of cellular experiments, we start the animal experiments. First, the PS@PDA‐ICG is injected to the subcutaneous tissue of model mice at the sites about 1 cm away from tumors. After injection, a tilted irradiation is applied to propel the PS@PDA‐ICG toward the designated tumor. By monitoring the propulsion process with NIR‐II imaging, we observe the NIR‐II fluorescence in the tumor after 9 min (Figure 5b) and the corresponding digital photos present a clear darkening of the tumor (Figure 5c). These results demonstrate an accumulation of PS@PDA‐ICG in the tumor. As depicted by the change of NIR‐II fluorescence intensity (Figure 5d), the accumulation gradually increases as the time prolonged. By contrast, for the control group without applying any irradiation, no appreciable fluorescence signal is detected in the tumor after 30 min or even 16 h (Figures 5e andS16, Supporting Information) and no significant change is found to the tumor color and fluorescence intensity (Figure 5f,g). The control experiments further highlight the irradiation‐enabled in vivo subcutaneous delivery.

With considerable PS@PDA‐ICG accumulated in the tumor, the PTT trials are performed. For trials, the mice bearing melanoma are randomly divided into a control group, a PS@PDA‐ICG group, and a PS@PDA‐ICG+NIR group. In the group with NIR, a favorable photothermal phenomenon is observed in the tumor (Figure 5h). Quantitative analysis shows that the tested temperature reaches up to approximately 60 °C after a 3 min irradiation (Figure 5i). Such high photothermal temperature can kill tumor cells and achieve the PTT efficacy. After performing the PTT, no significant changes are found on the body weight across all the groups (throughout a period of 21‐day observation, Figure 5j), indicating that the treatment feature minimal adverse effects to the mice. Quantitative analysis of tumor size in the PS@PDA‐ICG + NIR group finds a sustained reduction of tumor volume over time and the complete tumor disappearance occurs by the end of the observation period (Figure 5k–m). By contrast, the tumors in the other groups exhibit a progressive increase in size (Figure 5k–m). These results, together with the negligible therapeutic effect of the nanomotor itself, verify the in vivo PTT efficacy of the PS@PDA‐ICG.

After performing the PTT, we examine the pathological section of main organs (heat, liver, spleen, lung, kidney, Figure S17, Supporting Information). The pathological features of the mice treated with PS@PDA‐ICG are akin to that of the control, suggesting a negligible systemic toxicity for the PS@PDA‐ICG. To further clarify the systemic toxicity as well as immune response of PS@PDA‐ICG, the blood was collected through the eye socket for inflammatory cytokine test, hematology analysis, and serum biochemistry test. The indicators of the mice treated with PS@PDA‐ICG are very close to that of the control and all of them are in normal levels (Figures S18–S20, Supporting Information). The results of the above biological analysis show that the PS@PDA‐ICG features high biosafety and low immune response and could be further developed for in vivo applications.

## 3 Conclusion

In this work, we have developed a light‐powered integrated nanomotor for the efficient PTT of superficial tumors based on an ICG‐immobilized PS@PDA nanosphere. This motor can convert NIR‐I light into physical heat and NIR‐II fluorescence at the same time. The heat decays in the motor along the irradiation direction and exhibits threefold advantages. First, the heat attenuation creates a thermophoretic force toward the irradiation direction, providing a power for subcutaneous directed propulsion. Second, the heat itself can ablate adipose cells and protein fiber networks in the subcutaneous tissue, which reduces or even eliminates the resistance of subcutaneous propulsion. Third, the heat enables the PPT when the motors reach designated tumor sites. Meanwhile, the NIR‐II fluorescence can be harnessed for subcutaneous NIR‐II imaging via a commercial imaging system, enabling the real‐time tracking and directed propulsion of motors in subcutaneous tissue. Furthermore, the fabrication and functionalization (achieved by replacing the PS core) of our developed motor is straightforward, suitable for large‐scale production with high yield. To conclude, this work demonstrates an approach of superficial tumor PTT that has the potential to revolutionize currently‐available strategies, and suggests an integrated nanorobots and NIR‐II imaging‐based tracking method for this approach.

The in vivo imaging is crucial for biomedical applications of micro‐/nanomotors. It can guarantee precise navigation toward specified lesion sites. The reported imaging technologies include fluorescence imaging,^[^ ^33^ ^]^ photoacoustic imaging,^[^ ^57^ ^]^ ultrasound imaging,^[^ ^58^ ^]^ magnetic resonance imaging (MRI),^[^ ^26^, ^33^ ^]^ positron emission tomography (PET),^[^ ^44^ ^]^ and optical coherence tomography.^[^ ^40^ ^]^ The fluorescence imaging of them is featured with the advantages of real‐time imaging, easy operation, better specificity and higher sensitivity, and meantime, is challenged by its unsatisfactory penetration depth. As a kind of fluorescence imaging technology, NIR‐II imaging applied herein is naturally of much higher penetration depth compared to previously‐reported fluorescence imaging at visible light window, which offers a promising approach to resolve the challenge on penetration depth.^[^ ^59^, ^60^ ^]^ More importantly, the use of NIR‐I light as the excitation light makes the NIR‐II imaging easy to be integrated with NIR‐I powered propulsion and photothermal therapy. All the above advantages, brought together, suggest that the NIR‐II imaging is highly desired for superficial tumor PTT mediated by light‐powered nanomotors. Also, the application of NIR‐II imaging can be extended to other superficial disease treatment.

As suggested by Sylvain Martel et al.,^[^ ^26^ ^]^ living magneto‐aerotactic bacteria, a natural bio‐micromotor, could be developed as an active delivery agent for the superficial tumor treatment with peritumoral administration. Such bacteria can swim subcutaneous tissues, and attributed to its endogenous magnetosomes, the swimming can be easily controlled using magnetic fields. Furthermore, with the aid of an improved MRI machine, Sylvain Martel et al. have combined the magnetic field‐controlled swimming and MRI of the bacteria, achieving an MRI‐directed swimming in a mouse subcutaneous tissue. However, the survival and potential toxicity of magneto‐aerotactic bacteria remain challenging issues to their further developments. Different from living bacteria, our proposed motor uses external NIR‐I light for propulsion and thus can evade the survival issue of above. Regarding of the toxicity, our motor already shows high biocompatibility and it can be further improved by adjusting its chemical compositions (e.g., replacing the PS core with other materials). Besides, our motor is featured with easy fabrication, real‐time imaging and highly‐integrated functions. Nevertheless, the in vivo developments of our motor need to resolve several challenges including optimized propulsion in subcutaneous tissue, tumor penetration after reaching the tumor site, and integration of the thermophoretic propulsion, PTT and NIR‐II imaging on a commercial NIR‐II imaging system. Next, we will explore the optimized subcutaneous propulsion for tumors with varying sizes and depths, and meantime, extend our motor to accelerate the wound healing in superficial region.

## 4 Experimental Section

### Materials

PS nanospheres and sodium hydroxide (NaOH) were purchased from the Aladdin Inc., China. Dopamine hydrochloride (DA·HCl), Tris(hydroxymethyl)aminomethane Hydrochloride (Tris‐HCl, analytical grade), calcein acetoxymethyl ester (calcein‐AM) and propidium iodide (PI) were purchased from the Sigma‐Aldrich Inc., Germany. ICG was purchased from the Shanghai Yuanye Biotechnology Company, China. Isoflurane was sourced from the RWD Life Science, USA. Physiological saline was purchased from the Beijing Solarbio Life Science, China. Ultra‐pure water was produced by using a Barnstead Nanopure (18 mΩ) system. All received chemicals were directly used without further purification. The animals including nude mice used in the experiments were obtained from the Jiangsu GemPharmatech Biotechnology Co., China.

### Fabricating Process

The PS@PDA were fabricated according to the protocol reported in the literature.^[^ ^50^ ^]^ A 30 mg DA·HCl was dissolved in 10 mL Tris‐HCl solution, followed by the addition of 200 µL PS aqueous solution to the obtained mixture. To initiate the polymerization of DA, the pH of the mixture was adjusted to 8.5 by using 0.1 m NaOH. The reaction solution was continuously stirred for 12 h. The intermediate product (PS@PDA nanospheres) was collected from the reaction solution via centrifugation and washed three times with ultrapure water and ethanol. Then, this product was added to 4 mL 25 µg mL^−1^ ICG solution. After being stirred for 12 h, the PS@PDA‐ICG were separated via centrifugation and washed three times with distilled water.

### Characterization

SEM images were acquired on a Carl Zeiss Supra 55 scanning electron microscope. UV‐Vis‐NIR spectra were recorded by the PerkinElmer Lambda 750 UV–vis–NIR spectrophotometer. FTIR measurement was carried out on a Bruker Hyperion 3000 spectrometer based on the KBr pellet method. Photoluminescence measurements of samples dissolved in PBS solution were carried out on an Applied Nanofluorescence Spectrometer (USA). The size distribution of nanomotor was measured by using a Zetasizer Nano ZS90 dynamic light scattering. CLSM images were acquired on a Zeiss Axio‐Imager LSM‐800 Confocal Light Scanning Microscope. Thermal images were taken with a Fotric 225s infrared thermal imaging system and the related temperature was directly read from this system. The microscopic images of subcutaneous tissues, obtained from chicken leg in the cases with and without the nanomotors (10 µL, 1 mg mL^−1^), were recorded with the Nikon Eclipse 80i microscope.

### Weight Percentage of ICG and PDA

First of all, the absorbance spectra of ICG (Figure S21a, Supporting Information) was measured at the designated concentration (i.e., 0, 10, 20, 30 µg mL^−1^). The peak intensity at 780 nm was read and plotted against the corresponding ICG concentration. The obtained result was the standard curve of ICG (Figure S21b, Supporting Information). Next, the absorbance spectra of 2 mL ICG supernatant collected from the fabricating process (Figure S21c, Supporting Information) was measured. The peak intensity was 0.64047, corresponding to the 5.42 µg mL^−1^ ICG by using the standard curve. All collected ICG supernatant was 5 mL and the initially added ICG was 100 µg. It meant that the mass of ICG immobilized on the nanomotor was 72.88 µg. The number of the nanomotor (PS@PDA‐ICG) was 2.22 × 10^9^, read from a hemocytometer purchased from Sigma‐Aldrich Inc). The freeze‐dried mass of the PS@PDA‐ICG was 8.09 mg and the added PS was 5.00 mg. According to the data of above, the study calculated the mass of an individual PS@PDA‐ICG (3.64 × 10^−9^ mg) and the mass of ICG (3.2 × 10^−11^ mg), PDA (1.36 × 10^−9^ mg) and PS (2.27 × 10^−9^ mg) in an individual nanomotor. With these mass values, the weight percentage of ICG, PDA, and PS can be calculated to be 0.88%, 37.36%, and 61.76%.

### Photo‐Thermophoretic Propulsion

In the photo‐thermophoretic propulsion of an individual nanomotor, the PS@PDA‐ICG (10 µL, 0.1 mg mL^−1^, diameter: ca. 1 µm) was first added into a 10 µL PBS solution. Then the obtained mixture was carefully dropped onto a glass slide and covered with a coverslip. A Nikon Eclipse 80i microscope was used to observe and record the motion behaviors of the nanomotor and an 808 nm light (Hi‐Tech Optoelectronics Co., Ltd.) was applied for propulsion. The titled 808 nm NIR light with different intensity (0, 0.5, 1, 1.5, 2 W cm^−2^) were applied to propel the nanomotor. Except the tilted irradiation, the propulsion of multi‐body nanomotors was also carried out under a vertical irradiation. To obtain the velocity and trajectory, the recorded videos were analyzed using a PhysVis software. The sample size for the analysis was set to be 3. The MSD of the motor motion was calculated in a Matlab software. The propulsion of other samples used the same methods as described above.

### In Vitro NIR‐II Fluorescence Imaging

The in vitro NIR‐II imaging of the nanomotors was carried out in a 3D‐printed microchannel with a commercial NIR‐II imaging system (DB‐1, Teltec Semiconductor Pacific Ltd.). The 808 nm 1.5 W cm^−2^ NIR‐I light (Hi‐Tech Optoelectronics Co., Ltd.) was used for actuation. Prior to applying the NIR‐I light for actuation, a 1 mL 1 mg mL^−1^ nanomotor suspension was carefully injected to the middle of the channel. Then, the light was exerted and the NIR‐II signal of the channel was recorded. The images were exported from the imaging system. The fluorescent intensity in the images was directly read using the software ImageJ 2.1.0.

### Cytotoxicity Assay

The mouse embryonic fibroblasts cell (NIH3T3) was selected for cytotoxicity assay. A cell spreading was performed to ensure that the number of cells per well was 8000–10000 in a 96‐well plate. After a 24 h incubation, the cell‐adherent growth rate reached more than 85% and the cytotoxicity validation experiments were performed. The PS@PDA‐ICG nanomotors with different concentration (0, 25, 50, 100, 200, 400, and 600 mg mL^−1^) were sequentially added into 96‐well plates, and then, the treated cells were placed in a constant temperature incubator at 37 °C for 24 h. After the incubation, a 10 µL CCK‐8 was sequentially added into the 96‐well plates, and incubated for 20–30 min in the presence of enzyme markers. The absorbance was tested using a Multiskan FC microplate detector (Thermo Scientific Inc., USA) at 450 nm.

### In Vivo NIR‐II Fluorescence Imaging

The equipment for the in vivo experiment is the same as that in vitro. 20 µL of PS@PDA‐ICG nanomotors (1 mg/mL) were in vivo injected into mouse subcutaneous tissues and then observed directly without the application of NIR light or subject to vertical and titled NIR light actuation (1.5 W cm^−2^). The NIR‐II fluorescence images were recorded by a commercial NIR‐II imaging equipment. In this work, the experimental protocols involving animals were all approved by the Animal Care and Use Committee of Xiamen University (XMULA20200193) and were licensed by the Department of Science and Technology of Fujian, China.

### Traction Measurement of Subcutaneous Tissues

The mouse subcutaneous tissues injected with nanomotors were sliced into 10‐micron thick slices by a cryosectioning machine. The subcutaneous tissues were measured for traction before and after NIR light irradiation (1.5 W/cm^2^) using a NanoWizard 3 setup (JPK Instruments, Germany) at 22  C. A cantilever (k = 0.1 N/m, MLCT‐O10, Bruker, Germany) glued with a spherical polystyrene bead (20 µm in diameter, Sigma‐Aldrich) was used for the indentation experiment. Force‐distance curves were collected on grids of 50 × 50 µm and 4 × 4 measurement points. Three to five grids located at the center of each subcutaneous tissues were sampled. The resulting force‐distance curves were analyzed using a Hertzian model fit for the spherical indenter. The apparent elastic modulus (*E*) was determined by the JPK data processing software.

### Photothermal Ablation of Tumor Spheres

To assess the effect of antitumor spheres, U87 cells (2000 cells per well) were seeded in a 96‐well plate with 1.5% agarose covering the bottom to aggregate the cells. After incubation for 3 days, the cell spheres formed at the bottom and were taken out to put into a microchannel. The PS@PDA‐ICG nanomotors were injected into the tumor spheres. Subsequently, the tumor spheres were treated with 808 nm NIR irradiation for 10 min (1.5 W cm^−2^). Meanwhile, PS@PDA‐ICG nanomotors were injected into tumor sphere without NIR irradiation, and tumor sphere without PS@PDA‐ICG nanomotors under NIR irradiation were set as the control groups. After incubation for 30 min, the tumor spheres were stained with calcein‐AM and PI for 15 min. The fluorescent images were observed by CLSM.

### Modal Animal Testing

One million A875 cells were injected to subcutaneous tissue for establishing the melanoma‐bearing mouse model and the model tumor could be formed in 5 days. For treatment, a 20 µL PS@PDA‐ICG nanomotors (1 mg/mL) were injected at 1 cm near the model tumor. Guided by the real‐time NIR‐II imaging, the PS@PDA‐ICG nanomotors were propelled toward the tumor under 808 nm NIR light irradiation (1.5 W cm^−2^). After accomplishing the propulsion, the mice were treated with the same 808 nm NIR light (1.5 W cm^−2^) for PTT. Two control groups were also established and treated. One group did not use the PS@PDA‐ICG nanomotors and the other did not perform the PTT operation. The tumor size and mouse body weight were monitored each day. The tumor volume was calculated via volume = length × width^2^/2.

### Blood Analysis

In order to verify the biosafety of PS@PDA‐ICG in vivo. Healthy BALB/c mice (male, 6–8 weeks) were used in this experiment. The mice were divided into two groups (1) Saline, (2) PS@PDA‐ICG. The mice were intravenously injected with saline and PS@PDA‐ICG (20 mg kg^−1^ per mouse, Cur equivalent). After 3 days of administration, blood was collected through the eye socket for blood routine, serum biochemistry, and inflammatory cytokine tests.

### Tissue Histological Evaluation

The A875 tumor‐bearing BALB/c mice (male, 6–8 weeks) were euthanized after 7 days of treatment. Then, tumor, surrounding tissue of tumor, heart, liver, spleen, lung, and kidney tissues were fixed with a solution of 4% paraformaldehyde for 24 h. After that, these tissue sections were obtained and stained with H&E, Masson, immunofluorescence, or immunohistochemistry, respectively. Finally, the tissues were observed by CaseViewer scanner (Pannoramic SCAN).

## Conflict of Interest

The authors declare no conflict of interest.

## Author Contributions

J.J., J.H., and M.L. contributed equally to this work. X.H., M.S., and B.D. designed and supervised this research work. J.J., J.H., and M.L. conducted the experiments, analyzed the data, and wrote the manuscript. M.Z. conducted the traction measurement. All authors discussed the results and revised the manuscript.

## Supporting information



Supporting Information

Supporting Information

Supporting Information

Supporting Information

Supporting Information

## Data Availability

The data that support the findings of this study are available from the corresponding author upon reasonable request.
