# Supplementary material for: NIR‐II Fluorescent Thermophoretic Nanomotors for Superficial Tumor Photothermal Therapy
Source: Adv Mater. 2025 Feb 2;37(10):2417440. doi: 10.1002/adma.202417440 (PMC11899490; doi:10.1002/adma.202417440)
Supplement: Supplementary file 1 — Supporting Information [file ADMA-37-2417440-s004.pdf]

ADVANCED MATERIALS

Supporting Information

for *Adv. Mater.*, DOI 10.1002/adma.202417440

NIR-II Fluorescent Thermophoretic Nanomotors for Superficial Tumor Photothermal Therapy

Jiwei Jiang, Jing Hu, Mingtong Li, Mingzhi Luo, Bin Dong, Metin Sitti* and Xiaohui Yan**

Supplementary Materials for

NIR-II Fluorescent Thermophoretic Nanomotors for Superficial Tumor Photothermal Therapy

Jiwei Jiang[1]†, Jing Hu[2]†, Mingtong Li[3]†, Mingzhi Luo[5], Bin Dong[1*], Metin Sitti[3,4*], Xiaohui Yan[2*]

[1]Institute of Functional Nano & Soft Materials (FUNSOM), Jiangsu Key Laboratory for Carbon-Based Functional Materials & Devices, Soochow University, Suzhou 215123, China

[2]State Key Laboratory of Vaccines for Infectious Diseases, Center for Molecular Imaging and Translational Medicine, Xiang An Biomedicine Laboratory, School of Public Health, Xiamen University, Xiamen 361005, China

[3]Physical Intelligence Department, Max Planck Institute for Intelligent Systems, 70569 Stuttgart, Germany

[4]School of Medicine and College of Engineering, Koç University, 34450 Istanbul, Turkey

[5]Changzhou Key Laboratory of Respiratory Medical Engineering, Institute of Biomedical Engineering and Health Sciences, and School of Medical and Health Engineering, Changzhou University, Changzhou, Jiangsu, China

Supplementary Movies

Movie S1. The thermophoretic propulsion of an individual nanomotor in PBS under a tilted NIR-I irradiation. The video is recorded on a fluorescence microscope and speeded up three times using the VideoStudio software.

Movie S2. The thermophoretic propulsion of multi-body nanomotors in PBS under a vertical NIR-I irradiation. The video is recorded on a microscope and speeded up three times using the VideoStudio software.

Movie S3. The thermophoretic propulsion of multi-body nanomotors in PBS under a title NIR-I irradiation. The video is recorded on a microscope and speeded up three times using the VideoStudio software.

Movie S4. The thermophoretic propulsion of an individual nanomotor in an agarose hydrogel under a tilted NIR-I irradiation.

Supplementary Figures

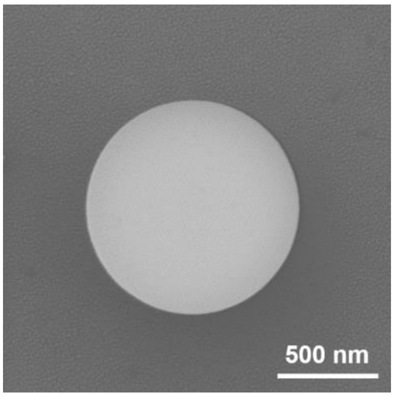

Figure S1. SEM image of a PS nanosphere.

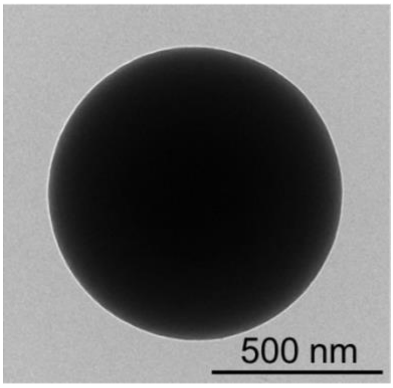

Figure S2. TEM image of a PS nanosphere.

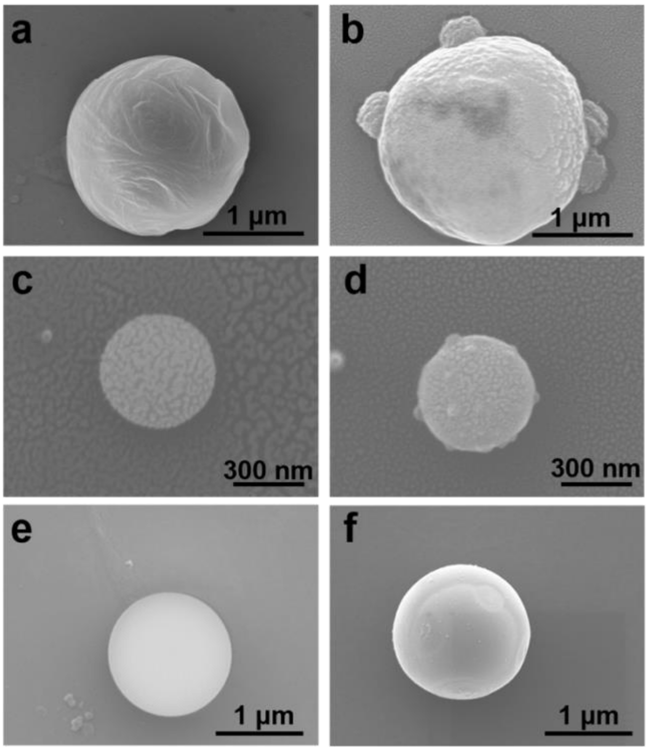

Figure S3. SEM images of the PDA-ICG composite deposited on three different cores. (a-b) The polycaprolactone (PCL) and PCL@PDA-ICG microsphere. (c-d) The magnetite (Fe_3O_4) and Fe_3O_4@PDA-ICG nanosphere. (e-f) The silica (SiO_2) and SiO_2@PDA-ICG nanosphere.

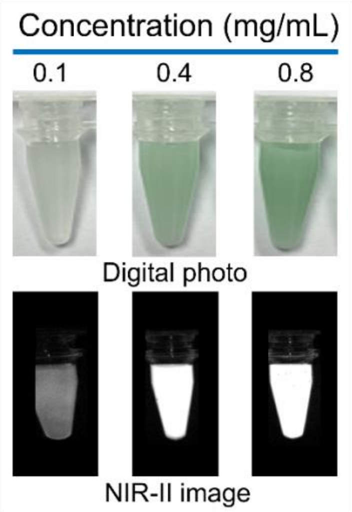

Figure S4. Digital photos and NIR-II images of 0.1, 0.4 and 0.8 mg/ml PS@PDA-ICG suspension.

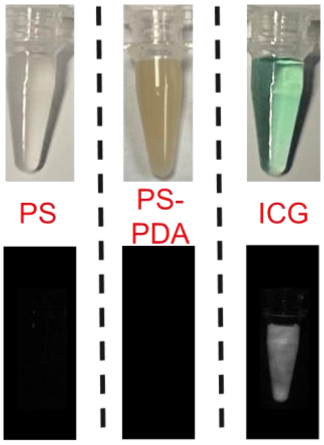

Figure S5. Digital photos and NIR-II images of the PS nanosphere suspension, PS@PDA nanosphere suspension and ICG solution.

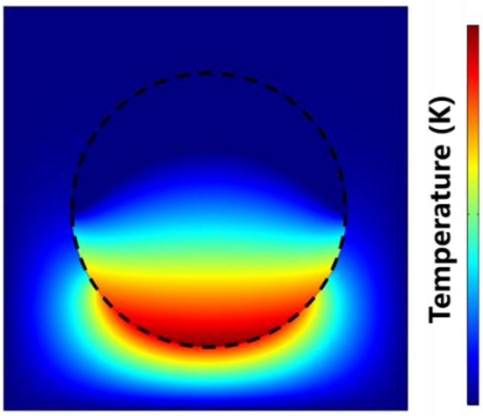

Figure S6. Simulation of the temperature distribution surrounding the PS@PDA-ICG irradiated by an 808 nm NIR light. The simulation was accomplished by using 2D models in the COMSOL Multiphysics 5.3 software package and a built-in nonlinear steady-state solver (MUMPS, multilateral parallel massively sparse direct solver).

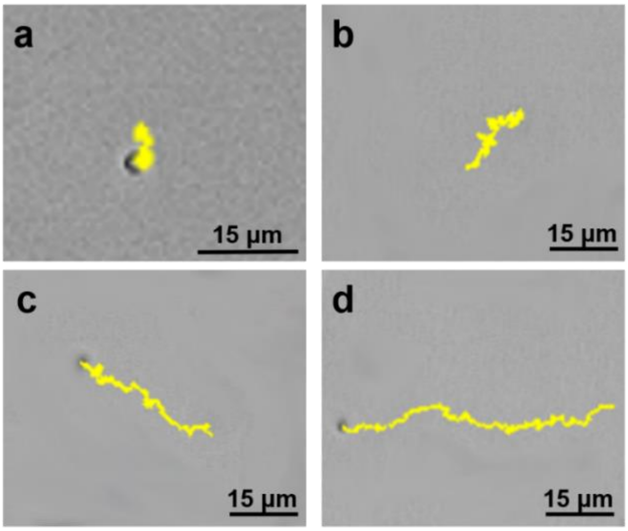

Figure S7. The motion trajectories of the PS@PDA-ICG nanomotor upon a 60 s NIR irradiation at the power of (a) 0 w/cm^2, (b) 0.5 w/cm^2, (c) 1 w/cm^2 and (d) 2 w/cm^2.

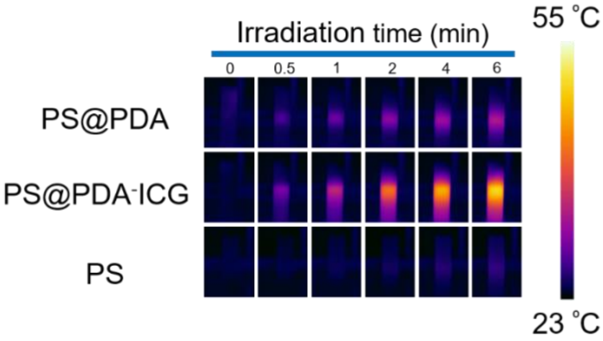

Figure S8. Time-lapse thermal images of 1 mg/mL PS, PS@PDA and PS@PDA-ICG subject to 1.5 W/cm^2 808 nm NIR-I irradiation.

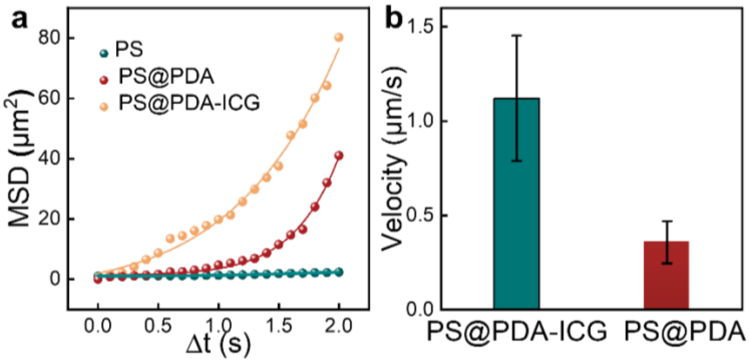

Figure S9. Motion behaviors of PS, PS@PDA and PS@PDA-ICG under a 1.5 W/cm^2 808 nm NIR irradiation. (a) Mean square displacement (MSD) *versus* time interval. (b) Velocity of the PS@PDA and PS@PDA-ICG. Error bars shown herein are the standard deviation of three independent experiments.

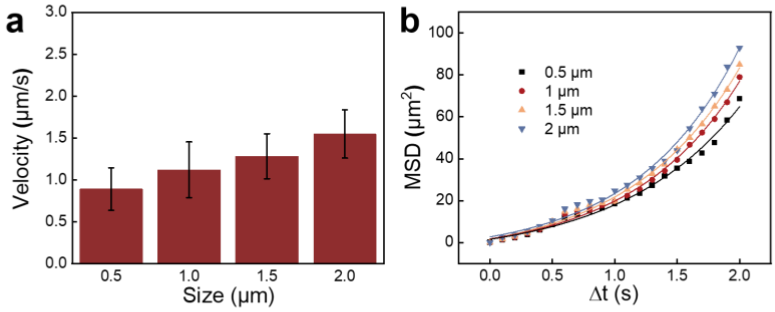

Figure S10. Motion behaviors of the PS@PDA-ICG at varying sizes. (a) The velocity *versus* different sizes. (b) Mean square displacement (MSD) *versus* time interval. Error bars shown herein are the standard deviation of three independent experiments. All samples are subject to the same NIR irradiation (1.5 W/cm^2, 808 nm).

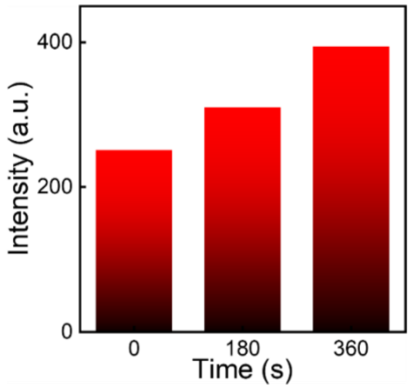

Figure S11. Change of the NIR-II signal intensity in Figure 4a.

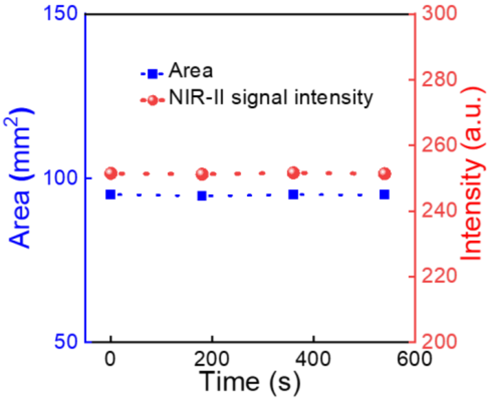

Figure S12. Changes of the NIR-II signal intensity and area in Figure 4c.

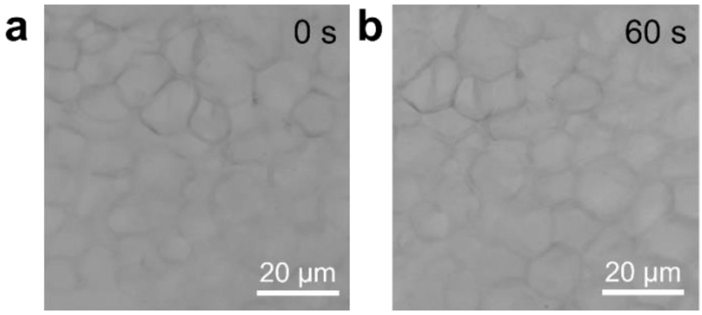

Figure S13. Adipose cells before and after a 60 s 1.5 W/cm^2 NIR-I irradiation.

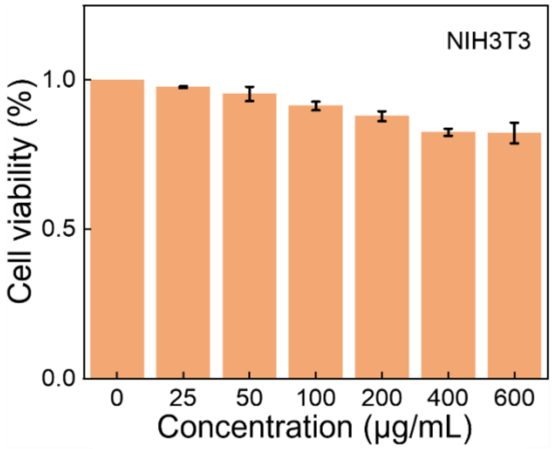

Figure S14. Cytotoxicity of the PS@PDA-ICG nanomotors to fiber cell line.

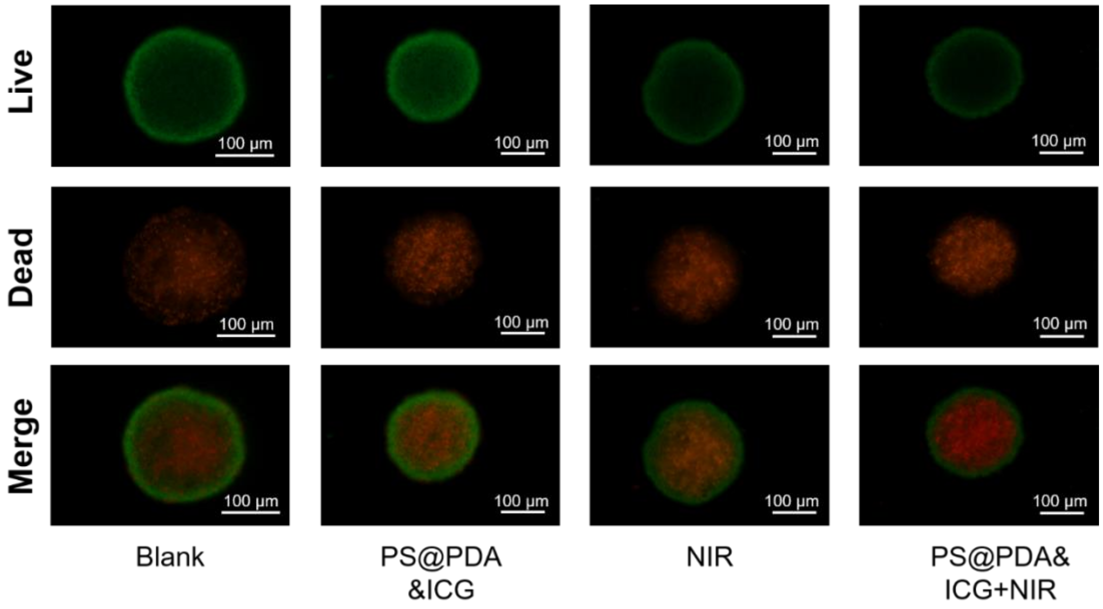

Figure S15. Fluorescence images of the tumor spheroids subject to different treatments and live/dead staining.

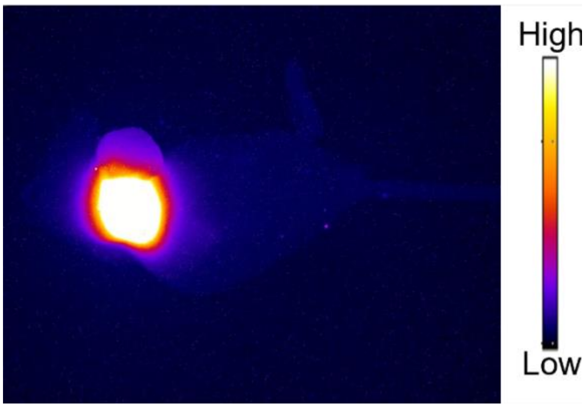

Figure S16. NIR-II fluorescence of a PS@PDA-ICG swarm acquired at 16 h after peritumor injection.

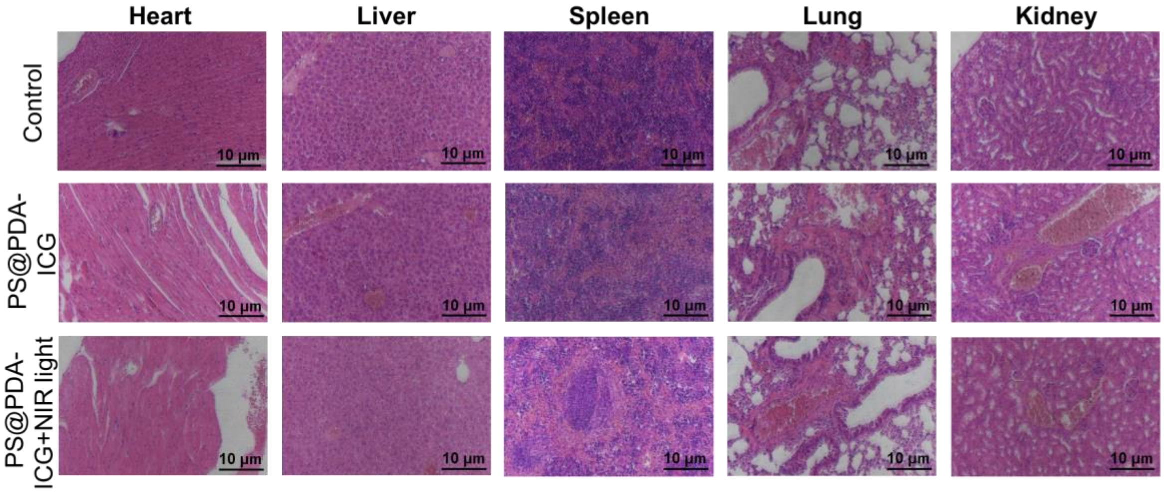

Figure S17. H&E-stained slices collected from different groups of mice after 21 days.

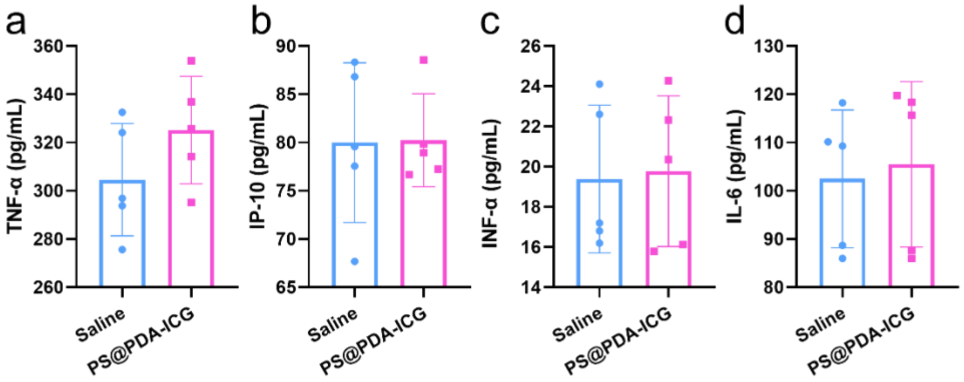

Figure S18. Plasma inflammatory cytokine levels in the mice treated with saline and PS@PDA-ICG nanomotors. (a) Tumor necrosis factor-α (TNF-α) levels. (b) Interferon g-inducible protein 10 (IP-10) levels. (c) Interferon α (IFN-α) levels. (d) Interleukin-6 (IL-6) levels.

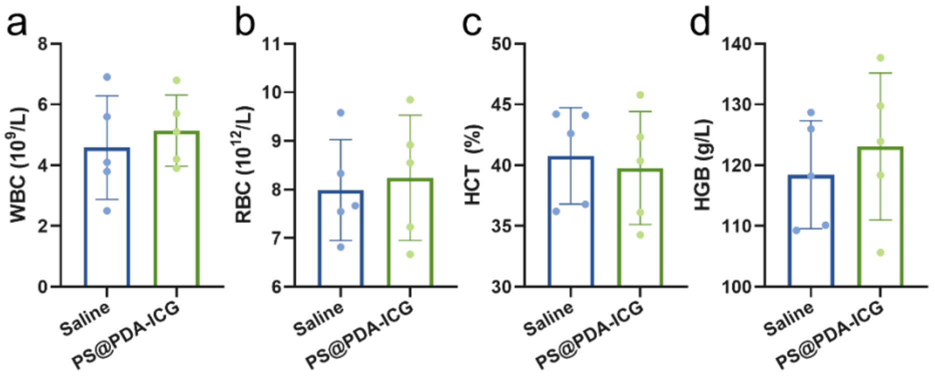

Figure S19. Hematology analysis in the mice treated with saline and PS@PDA-ICG nanomotors. (a) White blood cell (WBC) Concentration. (b) Red blood cells (RBC) concentration. (c) Hematocrit (HCT) concentration. (d) Hemoglobin (HGB) concentration.

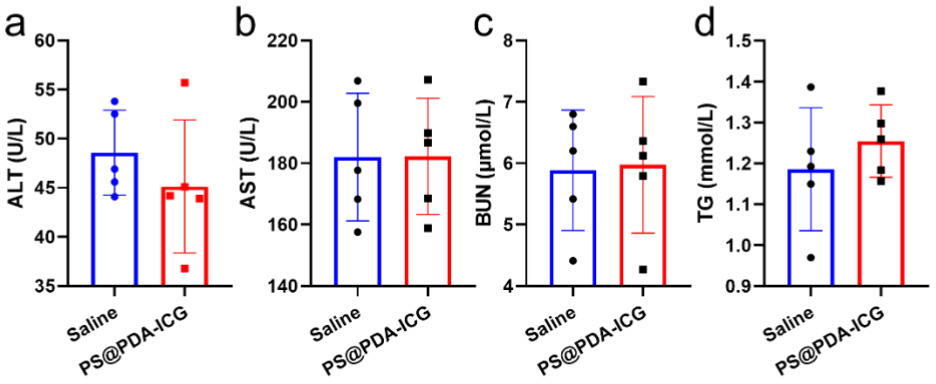

Figure S20. Serum biochemistry of the mice treated with saline and PS@PDA-ICG nanomotors. (a) Serum alanine aminotransferase (ALT) concentration. (b) Serum aspartate aminotransferase (AST) concentration. (c) Blood urea nitrogen (BUN) concentration. (d) Triglycerides (TG).

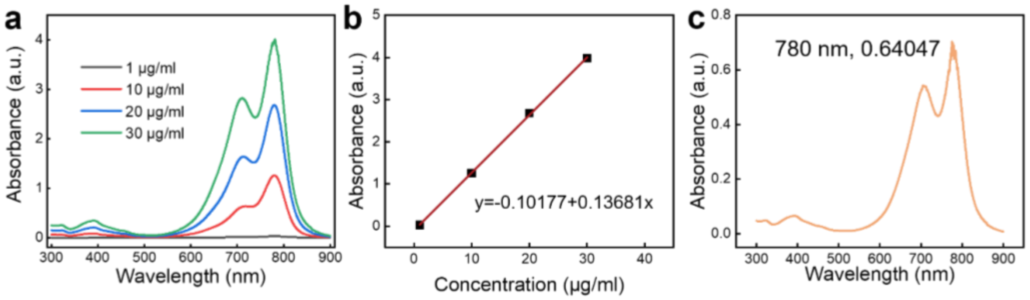

Figure S21. The absorbance spectra and standard curve of ICG. (a) The UV-vis-NIR spectra of ICG with varying concentrations. They are used for the plotting of the standard curve. (b) The standard curve of ICG. The absorbance intensity is the peak intensity at780 nm, directly read from (a). (c) The UV-vis-NIR spectrum of 2 ml ICG supernatant collected from the fabricating process.
